# Superoxide dismutases: marker in predicting reduced left ventricular ejection fraction in patients with type 2 diabetes and acute coronary syndrome

**DOI:** 10.1186/s12872-024-03867-2

**Published:** 2024-04-03

**Authors:** Xiu-Yun Jiang, Qing Chen, Xiao-Yu Chen, Qiu-Ying Sun, Fei Jing, Hai-Qing Zhang, Jin Xu, Xiao-Hong Li, Qing-Bo Guan

**Affiliations:** 1 Department of Endocrinology, Shandong Provincial Hospital Affiliated to Shandong First Medical University, Jinan, Shandong 250021 China; 2 International Medical Service Department, Shandong Provincial Hospital Affiliated to Shandong First Medical University, Jinan, Shandong 250021 China

**Keywords:** Superoxide dismutases, Patients with type 2 diabetes and acute coronary syndrome, Reduced left ventricular ejection fraction

## Abstract

**Aim:**

To examine the prognostic value of superoxide dismutase (SOD) activity for monitoring reduced left ventricular ejection fraction(LVEF)in the patients with type 2 diabetes and acute coronary syndrome (ACS).

**Methods:**

The population of this cross-sectional study included 2377 inpatients with type 2 diabetes who had an ACS admitted to the Shandong Provincial Hospital Affiliated to Shandong First Medical University from January 2016 to January 2021.

**Results:**

Diabetic patients with ACS were divided into 2 subgroups based on LVEF. The mean SOD activity was significantly lower in patients with an LVEF ≤ 45% than in those with an LVEF > 45% (149.1 (146.4, 151.9) versus 161.9 (160.8, 163.0)). Using ROC statistic, a cut-off value of 148.8 U/ml indicated an LVEF ≤ 45% with a sensitivity of 51.6% and a specificity of 73.7%. SODs activity were found to be correlated with the levels of NT-proBNP, hs-cTnT, the inflammatory marker CRP and fibrinogen. Despite taking the lowest quartile as a reference (OR 0.368, 95% CI 0.493–0.825, *P* = 0.001) or examining 1 normalized unit increase (OR 0.651, 95% CI 0.482–0.880, *P* = 0.005), SOD activity was found to be a stronger predictor of reduced LVEF than CRP and fibrinogen, independent of confounding factors.

**Conclusions:**

Our cross-sectional study suggests that SOD activity might be a valuable and easily accessible tool for assessing and monitoring reduced LVEF in the diabetic patients with ACS.

## Introduction

Patients with acute coronary syndrome (ACS) remain at high risk for recurrent cardiovascular events (CVEs) despite the use of guideline-recommended treatments. This risk is particularly high among patients with diabetes mellitus. The burden of cardiovascular events and death remains substantially high in patients with type 2 diabetes and established cardiovascular disease, even in patients with optimally controlled background risk factors and glycemic control [2]. Therefore, identifying prognostic biomarkers in diabetic patients with ACS is crucial for improving clinical management and reducing future CVEs. Various biomarkers and clinical factors were studied and found to be associated with worse outcomes in ACS patients [3–5]. However, there is still a need for an ideal biomarker that can be widely used with high accuracy to predict possible outcomes in clinical practice.

Declining left ventricular ejection fraction(LVEF)(below 45%) has been reported to be an important and powerful predictor of cardiovascular outcomes, including heart failure (HF) hospitalization, and cardiovascular mortality in the course of myocardial infarction [6–8]. Superoxide dismutase (SOD) has been studied to identify subgroups of patients at high risk of HF [9, 10], although cardiac troponins and natriuretic peptides are the most widely used predictive biomarkers in the management of HF [11]. Although a study has recently shown that SOD is a potential link between LV structural remodeling and the subsequent development of HF in patients with CVD [12], the relationship between SOD and LVEF has not been evaluated in the diabetic patients with ACS.

Therefore, the aim of this study was to examine the prognostic value of serum SOD activity for monitoring LVEF in the diabetic patients with ACS, particularly in the context of many other known risk factors for clinical outcomes within one population.

## Methods

### Study subjects

The study included 4302 inpatients with type 2 diabetes who had ACS admitted to Shandong Provincial Hospital Affiliated to Shandong First Medical University from January 2016 to January 2021. Patients could have T2DM diagnosed by the World Health Organization criteria before qualifying for ACS, and ACS was defined as an unstable angina, ST-segment elevation MI (STEMI), or non-STEMI [13]. The major exclusion criteria were (1) type 1 diabetes; (2) an age less than 30 years; (3) a previous history of percutaneous coronary intervention, coronary-artery bypass graft surgery, coronary revascularization procedures, uncontrolled arrhythmias, significant valve disease, renal dysfunction, liver problems, or all types of cancer; and (4) missing or incomplete echocardiography parameters, laboratory measurements, clinical characteristics, or demographic characteristics. Therefore, a total of 2377 subjects (1442 men and 935 women), in whom LVEF was available after ACS, were included. The proportion of cases including diabetes with unstable angina, STEMI, and non STEMI was 1854 (78%), 388 (16.3%), and 135 (5.7%), respectively.

Written informed consent was obtained from all subjects. The study was approved by the institutional review board of Shandong Provincial Hospital Affiliated to Shandong First Medical University. All methods were performed in accordance with the relevant guidelines and regulations.

### Clinical and laboratory assessment

Patients were managed according to the clinical framework of guideline based best medical treatment implemented by the institution. This framework includes measuring peak levels of cardiac biomarkers (troponin T and creatine phosphokinase) and routine clinical biomarkers (including total SOD activity) within 48 h of admission, as well as comprehensive echocardiography.

Echocardiography was performed by an experienced cardiologist. The LVEF was measured using a General Electric GE Vivid E9 Ultrasound System, and by the biplane method (Simpson) when the endocardial border of the left ventricle was well defined and whenever regional wall-motion abnormalities were present, or alternatively by the Teichholz method [14]. A LVEF ≤ 45% was defined as a reduced LVEF. Serum total SOD activity was measured by using the pyorgallol autoxidation method (Superoxide Dismutase Assay Kit, Fuyuan Biotechnology Co. Ltd., Fujian, China). The level of serum CRP was detected by the immunoturbidimetric method (Full Range C-Reactive Protein Reagent Kit, Dongou Biotechnology Co. Ltd., Zhejiang, China), following the manufacturer’s instructions using an automatic analyzer (Beckman Coulter Chemistry Analyzer AU5800, Beckman Coulter Co., Ltd, Tokyo, Japan). The level of serum fibrinogen was measured by a HemoslL Fibrinogen-C XL using a coagulation instrument (Werfen ACL TOP700, Instrumentation Laboratory Co., NY, USA). Baseline population characteristics were collected from medical records, prior medication and self-reports. All the data were managed and quality controlled with an electronic data capture system (Yiducloud Technologies Co., Ltd).

### Statistical analysis

The distribution of the different variables was examined for normality by the Kolmogorov-Smirnov test. Continuous variables were expressed as the mean (SD) or geometric mean (95% confidence interval) and categorical variables were expressed as percentages. Between-group differences with respect to continuous variables with a normal distribution were assessed using the Student’s t test or one-way ANOVA, and continuous variables with a non-normal distribution were assessed using the Mann–Whitney U test or Kruskal–Wallis test. Between-group differences with respect to categorical variables were assessed using a chi-square test.

Unadjusted and adjusted logistic regression analyses were performed to evaluate the intensity of the association between each biomarker and reduced LVEF. Adjusted odds ratio (OR) and 95% confidence interval (95% CI) were calculated. The variables were selected based on univariate analysis (*P* < 0.05). The model used in fully adjusted logistic regression analysis included gender, smoking history, systolic blood pressure (SBP), alanine aminotransferase (ALT), high density lipoprotein cholesterol (HDL-C), fasting blood glucose (FBG), HbA1c, serum creatinine (Cr), serum uric acid (UA), NT-proBNP and hs-cTnT. Age, diastolic blood pressure (DBP), aspartate aminotransferase (AST), low density lipoprotein cholesterol (LDL-C), total cholesterol (TC) and triglyceride (TG) were not included as biomarkers. The OR and 95% CI for reduced LVEF were examined by taking each biomarker as nominal and continuous variables, respectively. For nominal variables, the OR was calculated as the lowest quartile of each biomarker used as the reference. For continuous variate, each biomarker was normalized by the Z-score method to compare their predictive value, and the OR was subsequently examined by evaluating a normalized 1-unit increase.

The predictive value of each biomarker for reduced LVEF was assessed by receiver operating characteristic (ROC) analysis. The areas under the ROC curves (AUCs) were determined and then compared by the nonparametric Z-test. Youden’s index (sensitivity + specificity − 1) was used to determine the optimal cutoff point for each indicator. Data analyses were performed with SPSS Statistics (version 19.0). A P-value (two tailed) < 0.05 indicated statistical significance.

## Results

The baseline demographic and clinical characteristics of the 2377 diabetic patients with ACS included in this study are summarized in Table 1. The average age of all the participants was 63.0 (62.9, 63.6) years, and 1442 (60.7%) of them were male. Among all of the participants, the median LVEF was 60% (interquartile range 53–60%), 2185 (91.9%) patients had LVEF > 45%, and 192 (8.1%) patients had LVEF ≤ 45%. A significant decrease in serum SOD activity was observed in the patients with LVEF ≤ 45% compared to patients with LVEF > 45% (149.1 (146.4, 151.9) vs. 160.0 (159.0 161.1); *P* < 0.001). Compared with patients with LVEF > 45%, those with LVEF ≤ 45% were tended to have higher levels of CRP and fibrinogen. In addition, a higher SBP, AST, FBG, HbA1c, Cr, UA, NT-proBNP, and hs-cTnT and a greater proportion of male smokers, as well as a lower level of HDL-C and TG, were observed in patients with LVEF ≤ 45% (*P* < 0.01 for all). No significant differences were observed in age (*P* = 0.332), DBP (*P* = 0.112), ALT (*P* = 0.106), LDL-C (*P* = 0.972) or TC (*P* = 0.257) between the LVEF > 45% and LVEF ≤ 45% groups.

**Table 1:** Demographic characteristics and laboratory parameters of study participants

Characteristic		*Total population* (*n* = *2377)*	*With EF* > *45*% (*n* = *2185)*	*Patients with EF* ≤ *45*% (*n* = *192)*	P value
*Age (years)*		*63.0 (62.9, 63.6)*	*63.3(62.9 63.6)*	*62.9 (62.0, 63.8)*	*0.332*
*Gender (male-n-%)*		*1442 (60.7%)*	*1305 (59.7%)*	*137 (70.8%)*	*<0.001*
*Smoking (n-%)*		*995 (41.9%)*	*895 (41.0%)*	*100 (52.1%)*	*<0.001*
*Systolic blood pressure (mmHg)*		*137.1 (136.3, 137.9)*	*138.4 (137.6, 139.3)*	*128.9 (126.8, 131.0)*	*<0.001*
*Diastolic blood pressure (mmHg)*		*79.3 (78.8, 79.7)*	*79.3 (76.8, 79.8)*	*79.1 (77.6, 80.6)*	*0.112*
*ALT*		*24.42 (23.84, 24.99)*	*24.04 (23.43, 24.64)*	*26.65 (24.88, 28.42)*	*0.106*
*AST*		*24.61 (24.10, 25.12)*	*24.48 (23.93, 25.04)*	*25.38 (24.05, 26.70)*	*0.04*
*FBG (mmol/L)*		*8.36 (8.23, 8.49)*	*8.22 (8.12, 8.33)*	*8.72 (8.37, 9.07)*	*0.033*
*HbA1C(%)*		*7.92 (7.85, 7.99)*	*7.89 (7.81, 7.96)*	*8.16 (7.95, 8.38)*	*0.007*
*High-density lipoprotein (mmol/L)*		*1.08 (1.08, 1.10)*	*1.09 (1.07, 1.10)*	*1.04 (1.01, 1.06)*	*0.001*
*Low-density lipoprotein (mmol/L)*		*2.64 (2.61, 2.69)*	*2.64 (2.61, 2.68)*	*2.66 (2.57 2.75)*	*0.972*
*Total cholesterol (mmol/L)*		*4.31(4.26, 4.36)*	*4.32 (4.27, 4.37)*	*4.26 (4.14, 4.39)*	*0.257*
*Triglyceride (mmol/L)*		*1.83(1.77, 1.88)*	*1.85 (1.79, 1.79)*	*1.70 (1.53, 1.86)*	*<0.001*
*Serum creatinine (µmol/L)*		*66.83 (66.24, 67.42)*	*65.68 (65.06, 66.30)*	*73.63 (72.05, 75.21)*	*<0.001*
*Serum uric acid (µmol/L)*		*321.9 (328.3, 335.5)*	*323.3 (319.7, 326.9)*	*382.6 (371.4, 393.7)*	*<0.001*
*SOD (u/ml)*		*160.0 (159.0, 161.1)*	*161.9 (160.8, 163.0)*	*149.1 (146.4, 151.9)*	*<0.001*
*CRP (mg/L)*		*7.28 (6.55, 8.02)*	*6.44 (5.74, 7.15)*	*12.26 (9.38, 15.14)*	*<0.001*
*Fibrinogen (g/L)*		*3.37 (3.34, 3.41)*	*3.33 (3.29, 3.36)*	*3.64 (3.54, 3.75)*	*<0.001*
*NT-proBNP(pg/ml)*		*924.4 (843.3, 1005.6)*	*559.9 (510.2, 609.6)*	*3095.4(2679.8, 3511.0)*	*<0.001*
*Hs-cTnT (pg/ml)*		*157.4 (138.7, 176.1)*	*143.9 (124.4, 163.4)*	*237.8 (179.1, 396.5)*	*<0.001*

To determine the independent variables for the incidence of LVEF < 45%, multivariate logistic regression analysis was performed and the results are shown in Table 2. A significantly greater percentage of patients with an LVEF < 45% had lower SOD activity and higher levels of CRP and fibrinogen (*P* < 0.01 for all). After adjusting for gender, smoking habits, and systolic blood pressure, the associations between SOD activity, CRP, fibrinogen, and the incidence of LVEF < 45% did not change (Model 1). After further adjustments were made for ALT, HDL-C, FBG, HbA1c, Cr, UA, NT-proBNP and hs-cTnT, the associations between SOD activity and the incidence of LVEF < 45% continued to persist (Model 2), despite taking the lowest quartile as a reference (OR 0.368, 95% CI 0.493–0.825, *P* = 0.001) or examining 1 normalized unit increase (OR 0.651, 95% CI 0.482–0.880, *P* = 0.005). However, CRP (OR 1.076, 95% CI 0.846–1.368, *P* = 0.551 for taking the lowest quartile as a reference, OR 1.060, 95% CI 0.836–1.346, *P* = 0.63 for examining 1 normalized unit increase) and fibrinogen (OR 1.076, 95% CI 0.858–1.349, *P* = 0.528 for taking the lowest quartile as a reference, OR 1.082, 95% CI 0.855–1.368, *P* = 0.512 for examining 1 normalized unit increase) no longer had predictive implications for the incidence of LVEF < 45%.

**Table 2:** The correlation between SOD, CRP, fibrinogen and the reduced LVEF in diabetic patients with ACS

Biomakers	Variate type	No. EF < 45% Q1/Q2/Q3/Q4	No adjusted			Model 1			Model 2	
			OR (95% CI)	P-value		OR (95% CI)	P-value		OR (95% CI)	P-value
*SOD*	*Nomical* ^*a*^	*91/44/34/20*	*0.597 (0.517, 0.691)*	*<0.001*		*0.586 (0.504, 0.681)*	*<0.001*		*0.638 (0.493, 0.825)*	*0.001*
	*Continuous* ^*b*^		*0.534 (0.449, 0.635)*	*<0.001*		*0.519 (0.433, 0.621)*	*<0.001*		*0.651(0.482, 0.880)*	*0.005*
*CRP*	*Nomical* ^*a*^	*20/45/55/72*	*1.480 (1.287, 1.701)*	*<0.001*		*1.462 (1.268, 1.685)*	*<0.001*		*1.076 (0.846, 1.368)*	*0.551*
	*Continuous* ^*b*^		*1.261 (1.134, 1.403)*	*<0.001*		*1.218 (1.088, 1.363)*	*0.001*		*1.060 (0.836, 1.346)*	*0.63*
*Fibrinogen*	*Nomical* ^*a*^	*32/40/54/66*	*1.319 (1.152, 1.510)*	*<0.001*		*1.334 (1.162, 1.532)*	*<0.001*		*1.076 (0.858, 1.349)*	*0.528*
	*Continuous* ^*b*^		*1.379 (1.211, 1.569)*	*<0.001*		*1.324 (1.160, 1.512)*	*<0.001*		*1.082 (0.855, 1.368)*	*0.512*

Data are expressed as ORs (95% CI). No adjusted, simple logistic regression; Model 1, multiple logistic regression adjusted for gender, smoking habits, systolic blood pressure; Model 2, multiple logistic regression, using a forward stepwise procedure to select variables, further adjusted for alanine aminotransferase (ALT), high density lipoprotein cholesterol (HDL-C), fasting blood glucose (FBG), HbA1c, serum creatinine (Cr), serum uric acid (UA), NT-proBNP and hs-cTnT.

^a^ The OR was examined by regarding the lowest quartiles as reference; ^b^ The HR was examined by evaluating 1 normalized unit increase

**Fig. 1:**
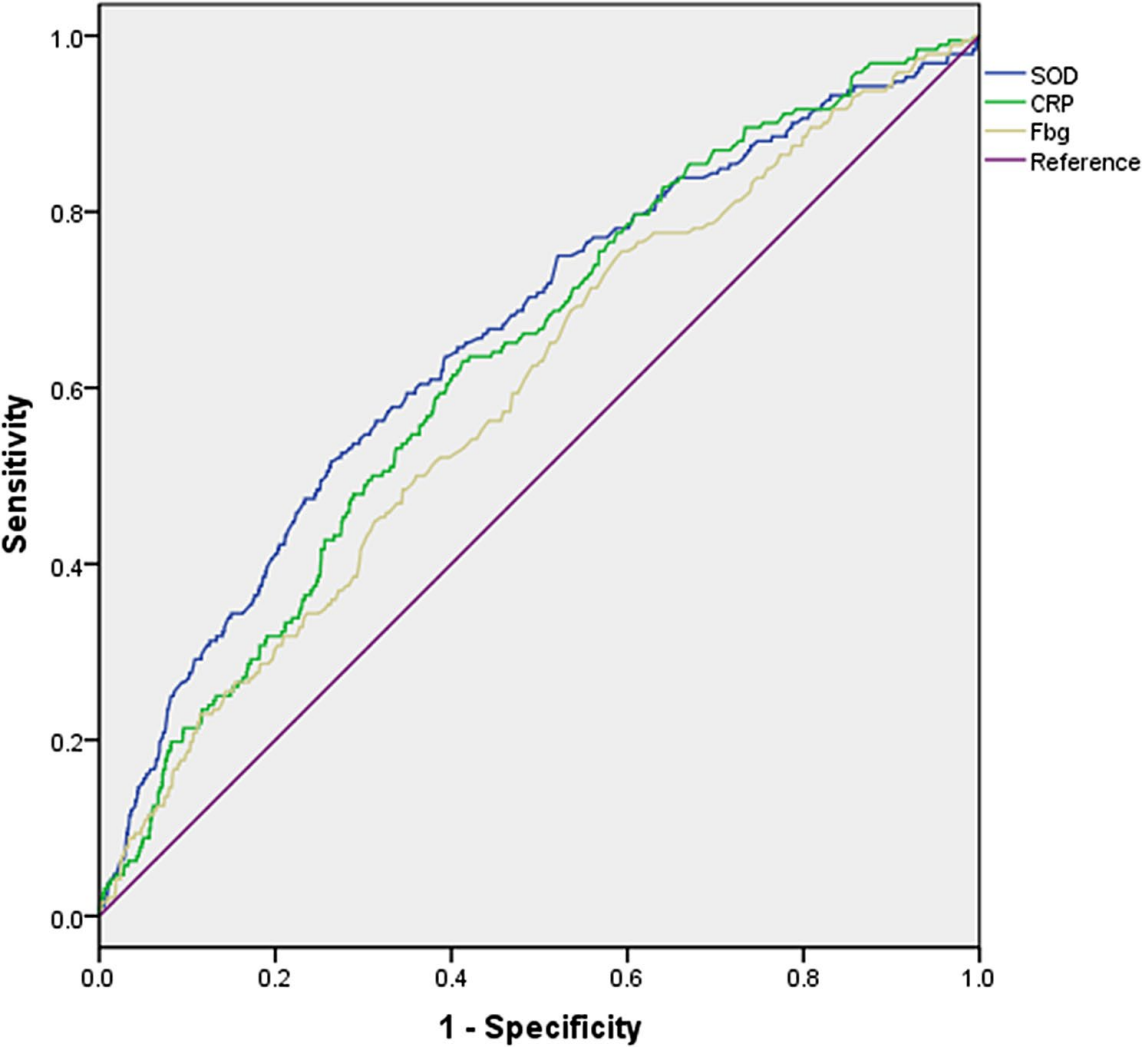
Receiver operating characteristic (ROC) curves of SOD, CRP and fibrinogen predicting incident of LVEF ≤ 45%

The accuracy of SOD and its sensitivity and specificity in correlating with an LVEF < 45% were compared, and the results are shown in Fig. 1; Table 3. The AUCs of SOD, CRP and fibrinogen were 0.658 (0.628, 0.688), 0.639 (0.613, 0.665) and 0.609 (0.528, 0.636) for an LVEF < 45%, respectively (P values for all < 0.001). Based on Youden’s index, the optimal cutoff values of SOD activity, CRP and fibrinogen for assessing the correlation with an LVEF < 45% were 148.8 U/ml, 2.56 g/L, and 3.05 g/L, respectively, with a sensitivity and specificity of 51.6% and 73.3%, 62.9% and 60.0%, and 74.6% and 42.8%, respectively. Further *Z* tests were conducted to compare the areas under the ROC curves. The results showed that there was a significant difference in the AUC between SOD and fibrinogen (Z = 2.234, *P* < 0.05). However, there was no significant difference in the AUC between SOD and CRP (Z = 0.896, *P* > 0.05), or between CRP and fibrinogen (Z = 1.368, *P* > 0.05).

**Table 3:** Areas under the ROC Curve (AUC), sensitivity and specificity by the optimized cutoff points for SOD, CRP and fibrinogen in assessing reduced LVEF

	AUC				Cutoff	Sensitivity (%)	Specificity (%)
	Est. (95% CI)	P-value	P-value for comparison*	P-value for comparison^#^			
*SOD (u/ml)*	*0.658 (0.628, 0.688)*	*< 0.001*	*-*	*> 0.05*	*148.8*	*51.6*	*73.7*
*CRP (mg/L)*	*0.639 (0.613, 0.665)*	*< 0.001*	*> 0.05*	*-*	*2.56*	*62.9*	*60*
*Fibrinogen (g/L)*	*0.609 (0.582, 0.636)*	*< 0.001*	*< 0.05*	*> 0.05*	*3.05*	*74.6*	*42.8*

## Discussion

The current study examined the correlation between SOD activity, CRP, fibrinogen, and reduced LVEF and compared their correlation with reduced LVEF in diabetic patients with ACS. The guidelines recommend that NT-proBNP be used in the predictive algorithm for HF [15], and hs-cTnT is an integral criterion in the diagnosis of AMI [16]. Our results demonstrated that compared with CRP and fibrinogen, SOD activity was the most relevant indicator of reduced LVEF after adjusting for hs-cTnT and NT-proBNP, in addition to other potential confounding factors (including gender, smoking status, systolic blood pressure, ALT, HDL-C, FBG, HbA1c, Cr, and UA). To our knowledge, this study is the first cross-sectional study to evaluate and compare the relationships between SOD, CRP, and fibrinogen levels and reduced LVEF in the diabetic patients with ACS.

SOD, as a major endogenous component of the antioxidant defense, is responsible for the inactivation of ROS in cardiomyocytes. Accumulating evidence derived from animal studies has demonstrated that SOD plays an important role in the development of HF. For example, previous studies have shown a significant decrease in SOD activity in rats with heart failure [17, 18]. Furthermore, gene-deficient mice lacking SOD and exposed to cardiac injury have demonstrated worse outcomes than wild-type mice [19], whereas mice overexpressing SOD and exposed to ischemia/reperfusion injury were found to have severely decreased levels of superoxide production, improved contractile function, and a decreased in infarct size [20]. Population studies have reported that the reduced SOD activity is closely associated with the increased vascular oxidative stress, which likely contributes to endothelial dysfunction in patients with HF [9]. The results from the most recent cross-sectional study showed that SOD activity is a potential link between left ventricular structural remodeling and the subsequent development of HF in patients with cardiovascular disease [12]. Our present study, it is further confirmed that SOD activity is associated with the reduced LVEF in the diabetic patients with ACS.

Molecular genetic studies have shown that a single-base substitution causing the exchange of glycine for arginine213 (Arg213Gly) in the heparin binding domain of SOD is associated with markedly increased plasma concentrations of the enzyme [9–11]. Previous studies have shown that SOD mutation is associated with excessive oxidative stress, endothelial dysfunction, and increased risk of ischemic heart disease [21, 22]. In the absence of mutations, increased SOD activity effectively protects against oxidative stress in tissues [23]. The serum SOD activity was normally distributed in our study, with a mean level of 160.0 (159.0, 161.1) U/mL, which is consistent with previously published data from other authors [12], suggesting that there were no carriers of *R213G* in our study population.

Patients with HF may manifest some of the clinical features observed in chronic inflammatory conditions [24]. CRP and fibrinogen are widely used inflammatory markers in routine clinical practice. Previous investigators have shown that CRP [25–28] and fibrinogen [29, 30] are correlated with cardiovascular events and HF independent of known cardiovascular factors. The results of a recent study showed that patients with higher CRP have features of more severe HF, and plasma CRP is independently related to subsequent mortality and morbidity [31]. However, the association between CRP and cardiovascular mortality in diabetes patients is controversial. Some researchers found that CRP was a significant predictor of cardiovascular disease only among individuals without diabetes [32–34]. In contrast, others have demonstrated that the association between CRP and cardiovascular mortality does not differ according to diabetes status [35–37]. The same is true for fibrinogen research. Although much positive evidence has been identified, the clinical significance of fibrinogen in the risk stratification of CVD patients is still controversial. For example, an analysis from the AtheroGene Study reported that the fibrinogen could not provide additional information to that provided by traditional cardiovascular risk factors in predicting cardiovascular events in adults without known cardiovascular disease [38]. However, the results from the Strong Heart Study showed that fibrinogen was strongly associated with incident HF in the cohort and this association persisted after adjusting for conventional risk factors [10]. The results from the present study showed that there is correlation between CRP and fibrinogen and reduced LVEF in diabetic patients with ACS. However, these correlations disappeared after a comprehensive logistic regression analysis of gender, smoking history, systolic blood pressure, ALT, HDL-C, FBG, HbA1c, Cr, UA, NT-proBNP and hs-cTnT. Recommendations regarding the use of CRP and fibrinogen in assessing the likelihood of reduced LVEF may need to be further reviewed.

Our results also showed that serum UA levels were significantly greater in patients with LVEF ≤ 45% than in patients with LVEF > 45%. The Endothelial dysfunction has been documented in coronary arteries in patients with HF [39]. A previous study revealed a positive correlation between UA levels, nitric oxide-mediated vasodilation and SOD activity in patients with HF [40]. Together with our finding that UA levels were within normal limits in the majority of the study patients, these findings suggested that increased UA levels could be part of an adaptive response to the increased oxidative stress present in the present study. Further experimental trials should be conducted to clarify the real impact of serum UA on the physiology of diabetic patients with ACS.

It has been reported that, despite common risk factors, men are predisposed to HF with a reduced LVEF. For example, among 2762 incident HF patients between 2000 and 2010 in Olmsted County [41], the proportion of men increased. Similarly, among 28 820 participants from four community-based cohorts followed for incident HF over a median follow-up of 12 years [42], men had an almost twofold greater risk of reduced LVEF than women. Together with our finding that a greater proportion of men were observed in diabetic patients with LVEF ≤ 45%, these findings illustrated that the most profound sex differences in cardiovascular medicine were found in heart failure patients [43].

The present study provides beneficial data for comparing the value of SOD activity and the levels of CRP and fibrinogen in assessing reduced LVEF in one report. In addition to showing that SOD activity could predict a reduced LVEF more than CRP or fibrinogen levels, the results also showed that there was no significant difference in the ability of CRP and fibrinogen to predict a reduced LVEF. This finding contrasts with reports that fibrinogen is more strongly associated with HF events than CRP in American Indians with a high incidence of obesity and diabetes [10]. Given the variability according to ethnicity, further studies are needed to assess the biomarkers in other populations.

Our study has several limitations. First, serum SOD activity was measured by using a commercial kit for measuring the enzyme activity of total SOD (including SOD1, SOD2 and SOD3 (extracellular SOD, EC-SOD)) in serum, which cannot be used to measure the activity of serum EC-SOD. However, total SOD activity is an inexpensive and widely used marker of antioxidant enzymes, and observing the changes in total SOD level can reveal the body damage and serve as an indicator of disease occurrence or an indicator of treatment efficacy. Second, the present findings were based on analyses using a historical cohort; however, the patients were consecutively added to the cohort. Third, we did not evaluate time-dependent changes in plasma SOD activity or CRP and fibrinogen levels during the treatment period. Fourth, the number of the study subjects was relatively small, therefore, the statistical power may be limited due to the small number of incident cases. Fifth, this study was carried out in a single urban university hospital with limited representation, which may not be representative of the entire Chinese population with diabetes and ACS.

In conclusion, the present study demonstrated that SOD activity and CRP and fibrinogen levels are correlated with reduced LVEF, moreover, SOD activity is the most relevant indicator of reduced LVEF in diabetic patients with ACS after adjusting for hs-cTnT and NT-proBNP, in addition to other potential confounding factors (including gender, smoking status, systolic blood pressure, alanine transaminase (ALT), high-density lipoprotein cholesterol (HDL-C), FBG, HbA1c, creatine (Cr), and uric acid (UA)). SOD activity combined with NT-proBNP and hs-cTnT may predict cardiovascular disease severity in diabetic patients with ACS.

## Data Availability

The management of all the data and quality control were performed with an electronic data capture system (Yiducloud Technologies Co., Ltd). The Data supporting the reported results are available from the corresponding author upon reasonable request.

## References

[1] Xiu-Yun Jiang QC, Chen X-Y, Sun Q-Y. Fei Jing, and 4 more. Association between Superoxide Dismutase, C-Reactive Protein, Fibrinogen and Heart Failure in Patients with Diabetes and Acute Coronary Syndrome. https://www.researchsquare.com/article/rs-1733313/v1.

[2] Rawshani A, Franzen S, Sattar N, Eliasson B, Svensson AM, Zethelius B (2018). Risk factors, mortality, and Cardiovascular outcomes in patients with type 2 diabetes. N Engl J Med.

[3] Karakayali M, Omar T, Artac I, Ilis D, Arslan A, Altunova M (2023). The prognostic value of HALP score in predicting in-hospital mortality in patients with ST-elevation myocardial infarction undergoing primary percutaneous coronary intervention. Coron Artery Dis.

[4] Morrow DA, Antman EM, Charlesworth A, Cairns R, Murphy SA, de Lemos JA (2000). TIMI risk score for ST-elevation myocardial infarction: a convenient, bedside, clinical score for risk assessment at presentation: an intravenous nPA for treatment of infarcting myocardium early II trial substudy. Circulation.

[5] Fox KA, Dabbous OH, Goldberg RJ, Pieper KS, Eagle KA, Van de Werf F (2006). Prediction of risk of death and myocardial infarction in the six months after presentation with acute coronary syndrome: prospective multinational observational study (GRACE). BMJ.

[6] Solomon SD, Anavekar N, Skali H, McMurray JJ, Swedberg K, Yusuf S (2005). Influence of ejection fraction on cardiovascular outcomes in a broad spectrum of heart failure patients. Circulation.

[7] Chew DS, Heikki H, Schmidt G, Kavanagh KM, Dommasch M, Bloch Thomsen PE (2018). Change in Left Ventricular Ejection Fraction following first myocardial infarction and outcome. JACC Clin Electrophysiol.

[8] Yildiz I, Rencuzogullari I, Karabag Y, Karakayali M, Artac I, Gurevin MS (2022). Predictors of left ventricular ejection function decline in young patients with ST-segment elevation myocardial infarction. Rev Assoc Med Bras (1992).

[9] Landmesser U, Spiekermann S, Dikalov S, Tatge H, Wilke R, Kohler C (2002). Vascular oxidative stress and endothelial dysfunction in patients with chronic heart failure: role of xanthine-oxidase and extracellular superoxide dismutase. Circulation.

[10] Barac A, Wang H, Shara NM, de Simone G, Carter EA, Umans JG (2012). Markers of inflammation, metabolic risk factors, and incident heart failure in American indians: the strong heart study. J Clin Hypertens (Greenwich).

[11] Wang XY, Zhang F, Zhang C, Zheng LR, Yang J (2020). The biomarkers for Acute myocardial infarction and heart failure. Biomed Res Int.

[12] Li X, Lin Y, Wang S, Zhou S, Ju J, Wang X (2020). Extracellular superoxide dismutase is Associated with Left Ventricular geometry and heart failure in patients with Cardiovascular Disease. J Am Heart Association.

[13] Bentley-Lewis R, Aguilar D, Riddle MC, Claggett B, Diaz R, Dickstein K (2015). Rationale, design, and baseline characteristics in evaluation of LIXisenatide in Acute Coronary Syndrome, a long-term cardiovascular end point trial of lixisenatide versus placebo. Am Heart J.

[14] Schiller NB, Shah PM, Crawford M, DeMaria A, Devereux R, Feigenbaum H (1989). Recommendations for quantitation of the left ventricle by two-dimensional echocardiography. American Society of Echocardiography Committee on standards, Subcommittee on quantitation of two-Dimensional echocardiograms. J Am Soc Echocardiography: Official Publication Am Soc Echocardiography.

[15] Ponikowski P, Voors AA, Anker SD, Bueno H, Cleland JGF, Coats AJS (2016). 2016 ESC guidelines for the diagnosis and treatment of acute and chronic heart failure: the Task Force for the diagnosis and treatment of acute and chronic heart failure of the European Society of Cardiology (ESC)developed with the special contribution of the Heart Failure Association (HFA) of the ESC. Eur Heart J.

[16] Roffi M, Patrono C, Collet JP, Mueller C, Valgimigli M, Andreotti F (2016). 2015 ESC guidelines for the management of acute coronary syndromes in patients presenting without persistent ST-segment elevation: Task Force for the management of Acute Coronary syndromes in patients presenting without Persistent ST-Segment Elevation of the European Society of Cardiology (ESC). Eur Heart J.

[17] Hill MF, Singal PK (1996). Antioxidant and oxidative stress changes during heart failure subsequent to myocardial infarction in rats. Am J Pathol.

[18] Khaper N, Kaur K, Li T, Farahmand F, Singal PK (2003). Antioxidant enzyme gene expression in congestive heart failure following myocardial infarction. Mol Cell Biochem.

[19] van Deel ED, Lu Z, Xu X, Zhu G, Hu X, Oury TD (2008). Extracellular superoxide dismutase protects the heart against oxidative stress and hypertrophy after myocardial infarction. Free Radic Biol Med.

[20] Wang P, Chen H, Qin H, Sankarapandi S, Becher MW, Wong PC (1998). Overexpression of human copper, zinc-superoxide dismutase (SOD1) prevents postischemic injury. Proc Natl Acad Sci USA.

[21] Iida S, Chu Y, Weiss RM, Kang YM, Faraci FM, Heistad DD (2006). Vascular effects of a common gene variant of extracellular superoxide dismutase in heart failure. Am J Physiol Heart Circ Physiol.

[22] Juul K, Tybjaerg-Hansen A, Marklund S, Heegaard NH, Steffensen R, Sillesen H (2004). Genetically reduced antioxidative protection and increased ischemic heart disease risk: the Copenhagen City Heart Study. Circulation.

[23] Chu Y, Alwahdani A, Iida S, Lund DD, Faraci FM, Heistad DD (2005). Vascular effects of the human extracellular superoxide dismutase R213G variant. Circulation.

[24] Katz AM, Katz PB (1962). Diseases of the heart in the works of Hippocrates. Br Heart J.

[25] Biasucci LM, Liuzzo G, Grillo RL, Caligiuri G, Rebuzzi AG, Buffon A (1999). Elevated levels of C-reactive protein at discharge in patients with unstable angina predict recurrent instability. Circulation.

[26] Ridker PM, Rifai N, Rose L, Buring JE, Cook NR (2002). Comparison of C-reactive protein and low-density lipoprotein cholesterol levels in the prediction of first cardiovascular events. N Engl J Med.

[27] Ridker PM, Cannon CP, Morrow D, Rifai N, Rose LM, McCabe CH (2005). C-reactive protein levels and outcomes after statin therapy. N Engl J Med.

[28] de Beer FC, Hind CR, Fox KM, Allan RM, Maseri A, Pepys MB (1982). Measurement of serum C-reactive protein concentration in myocardial ischaemia and infarction. Br Heart J.

[29] Palmieri V, Celentano A, Roman MJ, de Simone G, Best L, Lewis MR (2003). Relation of fibrinogen to cardiovascular events is independent of preclinical cardiovascular disease: the strong heart study. Am Heart J.

[30] Bahrami H, Bluemke DA, Kronmal R, Bertoni AG, Lloyd-Jones DM, Shahar E (2008). Novel metabolic risk factors for incident heart failure and their relationship with obesity: the MESA (multi-ethnic study of atherosclerosis) study. J Am Coll Cardiol.

[31] Anand IS, Latini R, Florea VG, Kuskowski MA, Rector T, Masson S (2005). C-reactive protein in heart failure: prognostic value and the effect of valsartan. Circulation.

[32] Sakkinen P, Abbott RD, Curb JD, Rodriguez BL, Yano K, Tracy RP (2002). C-reactive protein and myocardial infarction. J Clin Epidemiol.

[33] Best LG, Zhang Y, Lee ET, Yeh JL, Cowan L, Palmieri V (2005). C-reactive protein as a predictor of cardiovascular risk in a population with a high prevalence of diabetes: the strong heart study. Circulation.

[34] Biasucci LM, Liuzzo G, Della Bona R, Leo M, Biasillo G, Angiolillo DJ (2009). Different apparent prognostic value of hsCRP in type 2 diabetic and nondiabetic patients with acute coronary syndromes. Clin Chem.

[35] Kaptoge S, Di Angelantonio E, Lowe G, Pepys MB, Thompson SG, Collins R (2010). C-reactive protein concentration and risk of coronary heart disease, stroke, and mortality: an individual participant meta-analysis. Lancet.

[36] Kengne AP, Batty GD, Hamer M, Stamatakis E, Czernichow S (2012). Association of C-reactive protein with cardiovascular disease mortality according to diabetes status: pooled analyses of 25,979 participants from four U.K. prospective cohort studies. Diabetes Care.

[37] Lofblad L, Hov GG, Asberg A, Videm V (2021). Inflammatory markers and risk of cardiovascular mortality in relation to diabetes status in the HUNT study. Sci Rep.

[38] Sinning JM, Bickel C, Messow CM, Schnabel R, Lubos E, Rupprecht HJ (2006). Impact of C-reactive protein and fibrinogen on cardiovascular prognosis in patients with stable angina pectoris: the AtheroGene study. Eur Heart J.

[39] Hornig B, Arakawa N, Kohler C, Drexler H (1998). Vitamin C improves endothelial function of conduit arteries in patients with chronic heart failure. Circulation.

[40] Alcaino H, Greig D, Chiong M, Verdejo H, Miranda R, Concepcion R (2008). Serum uric acid correlates with extracellular superoxide dismutase activity in patients with chronic heart failure. Eur J Heart Fail.

[41] Gerber Y, Weston SA, Redfield MM, Chamberlain AM, Manemann SM, Jiang R (2015). A contemporary appraisal of the heart failure epidemic in Olmsted County, Minnesota, 2000 to 2010. JAMA Intern Med.

[42] Ho JE, Enserro D, Brouwers FP, Kizer JR, Shah SJ, Psaty BM et al. Predicting Heart failure with preserved and reduced ejection fraction: the international collaboration on heart failure subtypes. Circulation Heart Fail. 2016;9(6). Epub 2016/06/09. 10.1161/CIRCHEARTFAILURE.115.003116 PMC4902276 27266854

[43] Lam CSP, Arnott C, Beale AL, Chandramouli C, Hilfiker-Kleiner D, Kaye DM et al. Sex differences in heart failure. European heart journal. 2019;40(47):3859-68c. Epub 2019/12/05. 10.1093/eurheartj/ehz835 31800034

